# Using Virtual Reality in the Treatment of Gambling Disorder: The Development of a New Tool for Cognitive Behavior Therapy

**DOI:** 10.3389/fpsyt.2017.00027

**Published:** 2017-02-24

**Authors:** Stéphane Bouchard, Geneviève Robillard, Isabelle Giroux, Christian Jacques, Claudie Loranger, Manon St-Pierre, Maxime Chrétien, Annie Goulet

**Affiliations:** ^1^Université du Québec en Outaouais, Gatineau, QC, Canada; ^2^Centre Intégré de Santé et de Services Sociaux de l’Outaouais, Gatineau, QC, Canada; ^3^Université Laval, Québec, QC, Canada

**Keywords:** gambling disorder, virtual reality therapy, cognitive behavior therapy, cravings, craving behavior intervention, cognitive restructuring, side effect, safety

## Abstract

Virtual reality (VR) can be used in the treatment of gambling disorder to provide emotionally charged contexts (e.g., induce cravings) where patients can practice cognitive behavior therapy (CBT) techniques in the safety of the therapist’s office. This raises practical questions, such as whether the cravings are sufficient to be clinically useful but also manageable enough to remain clinically safe. Pilot data are also needed to test the development of a treatment manual and prepare large randomized control trials. This paper reports on three studies describing (a) cravings induced in VR compared to real gambling and a control game of skill with no money involved (*N* = 28 frequent gamblers and 36 infrequent gamblers); (b) the usefulness of a treatment protocol with only two CBT sessions using VR (*N* = 34 pathological gamblers); and (c) the safety of a four-session treatment program of CBT in VR (*N* = 25 pathological gamblers). Study 1 reveals that immersions in VR can elicit desire and a positive anticipation to gamble in frequent gamblers that are (a) significantly stronger than for infrequent gamblers and for playing a control game of skill and (b) as strong as for gambling on a real video lottery terminal. Study 2 documents the feasibility of integrating VR in CBT, its usefulness in identifying more high-risk situations and dysfunctional thoughts, how inducing cravings during relapse prevention exercises significantly relates to treatment outcome, and the safety of the procedure in terms of cybersickness. Results from Study 3 confirm that, compared to inducing urges to gamble in imagination, using VR does not lead to urges that are stronger, last longer, or feel more out of control. Outcome data and effect sizes are reported for both randomized control pilot trials conducted in inpatient settings. Suggestions for future research are provided, including on increasing the number of VR sessions in the treatment program.

People who suffer from gambling disorder (GD) are characterized by the inability to resist the urge to gamble, adversely affecting all aspects of their lives including their home, social, professional, and personal life (1). Cognitive behavior therapy (CBT) has repeatedly proven effective for this disorder. It constitutes an empirically validated form of treatment recommended by experts and is considered to be among best practices (2–5). Clinically, all of the founding literature on CBT [e.g., Ref. (6–9)] strongly emphasizes the importance of mastering therapeutic tools in the comfort of the therapist’s office and gradually transferring what is learned in the clinical setting to everyday situations of increasing difficulty. The literature review by Ledgerwood and Petry (10) identified this transfer of skills from the therapist’s office to a real-life context as important to prevent relapses in individuals suffering from GD. The lack of correlation between the place where CBT takes place—i.e., the therapist’s office—and the day-to-day reality of gamblers becomes particularly evident when it comes to the cravings and emotional responses felt by people suffering from addictive disorders when they encounter high-risk situations (3, 11).

There have been various attempts in CBT to help gamblers practice therapeutic strategies in situations of emotional arousal and cravings to gamble (3). For example, research has been done on the effectiveness of imaginal exposure (12, 13), where gamblers picture a high-risk situation in their minds so that they can then imagine themselves using psychotherapeutic strategies to deal with it. The few studies on this topic suggest that imaginal exposure helps reduce cravings in pathological gamblers (12, 14, 15). But, this technique is still limited because (a) not everyone is skilled at bringing the stimuli to life in their mind; (b) therapists have no way of knowing what, exactly, their clients are thinking about during the exercises; (c) the therapeutic exercises performed by clients increase their cognitive load, causing a corresponding decline in their ability to fully imagine themselves in that situation; and (d) therapists sometimes have trouble getting gamblers to put their dysfunctional thoughts into words.

Furthermore, inciting an urge to gamble by thinking of a past situation is still very limited when comparing an imaginary situation to the omnipresence and abundance of indicators capable of triggering a craving in the everyday lives of people who suffer from GD. There are many factors that contribute to high-risk situations for gamblers (16), such as images and logos associated with gambling, being in the presence of video lottery terminals (VLTs) in public places, feeling strong physiological and affective responses, seeing others gamble and win or give up their spot because they lost, being in the relaxed atmosphere of a bar, or the glamorous surroundings of a casino, etc.

Practising CBT in virtual reality (VR) offers a promising alternative (11, 17–20). In the safety of the therapist’s office, gamblers can don 3D glasses and be faced with VLTs or visit a casino. The therapist can then conduct various classic CBT interventions [see, for example, Ref. (21)], gradually bringing gamblers into situations that will trigger their urge to gamble. As described in Study 2 and 3 later in this article, therapists can use VR to identify situations, thoughts, and behaviors associated with gambling; conduct cognitive restructuring with dysfunctional beliefs underlying GD; or work on relapse prevention (18). Using VR in combination with traditional CBT has proven effective in a few studies, but these studies are all based on the therapeutic rationale of cue exposure [i.e., habituation leading to extinction of the conditioned response (19, 20)] and not on the goal of inducing emotions and cravings to practice CBT techniques (18, 22). Also, because the latter paradigm is not based on extinction, it raises the very important question of whether the cravings induced by VR are too strong to be used safely, especially in outpatient settings where people can go gamble after the therapy session.

The goal of this paper is to document the potential of VR in the CBT of GD with three consecutive studies that are as follows: (a) an experimental demonstration that VR immersions can induce cravings in GD patients (Study 1); (b) a pilot study documenting the potential of a minimal use of VR in CBT for GD (Study 2); and (c) a second pilot trial to gage the safety, in terms of the intensity of cravings, of a slightly more intensive use of VR in CBT for GD (Study 3). Due to ethical considerations regarding the induction of cravings in patients, the first study was conducted with a subclinical sample, the latter two studies were conducted in inpatient settings, and the number of sessions using VR progressively increased from only two in Study 2 to only four in Study 3. All three studies were approved by UQO’s review board of ethical conduct in research, and every participant signed an informed consent form in accordance with Canadian standards of ethical conduct for research involving human participants.

## Study 1

### Participants

To test whether the virtual environments developed for GD can induce an urge to gamble, adults between the ages of 18 and 65 familiar with VLTs were recruited. The sample included 28 “frequent” players (play VLTs at least once a month) and 36 “occasional” recreational players (play no more than twice a year). Occasional recreational gamblers were recruited as a control group with enough minimal experience with VLTs to know what they are and how to use them. Occasional recreational players were excluded if they scored higher than 1 on the South Oaks Gambling Screen (SOGS). Frequent players were excluded if they scored 9 or more for ethical reasons due to concern about inducing cravings in people suffering from GD [9 is a score clearly within the range of potential GD but below the severity of the majority of those diagnosed with GD (23, 24), p. 11]. Hypersensitivity to cybersickness, defined as a self-reported history of severe motion sickness when calling potential study participants, was also an exclusion criterion. Two additional exclusion criteria were set *a priori* and tested in the lab once the consent form was filled out, having poor stereoscopic vision or being intoxicated during the experiment, but no participant was excluded based on these criteria.

### Method

**Figure 1:**
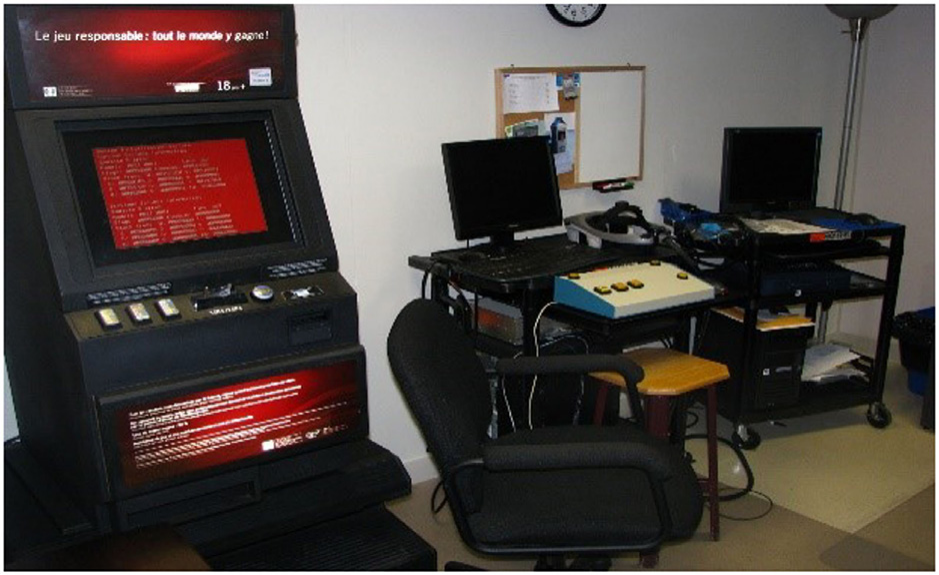
**Experimental setup for Study 1 on the potential to induce cravings with a real video lottery terminal and virtual reality**.

**Figure 2:**
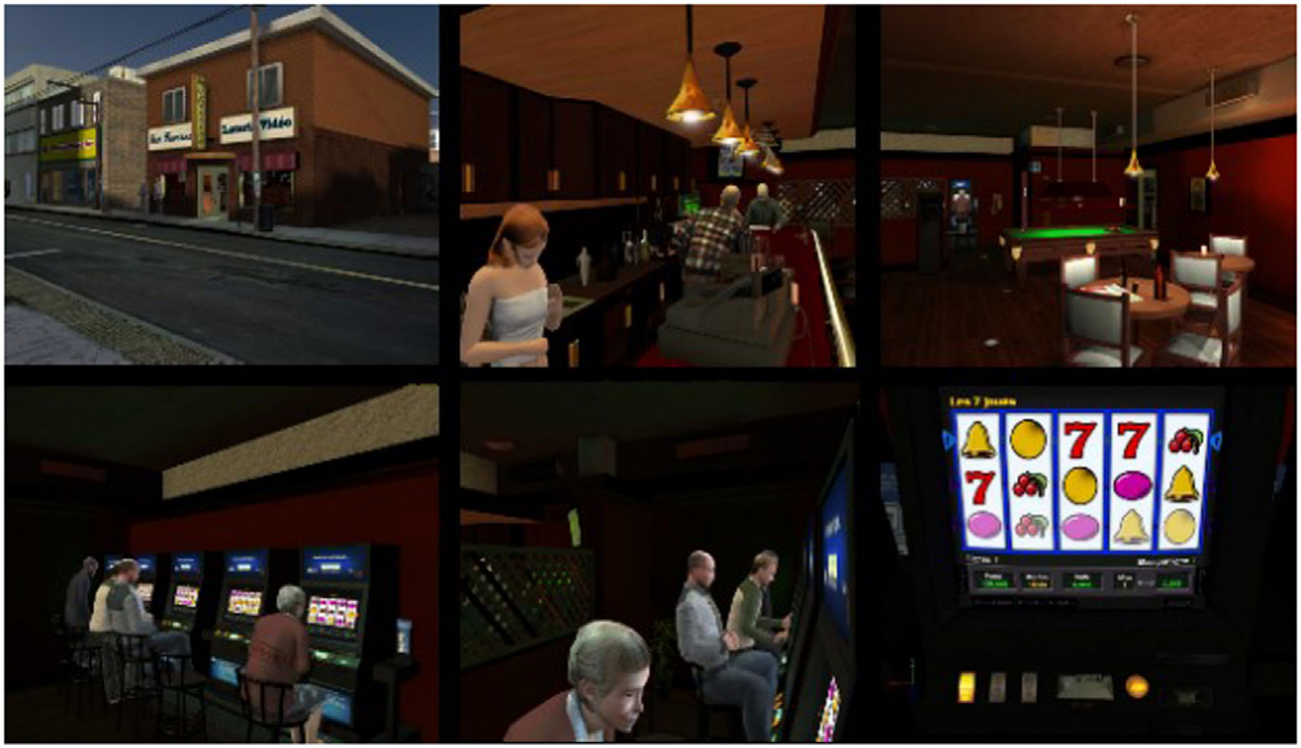
**Illustrations (screenshots) of the *At Fortunes* virtual environment used in all three studies**. Reproduced from Bouchard et al. (18) under the Creative Commons copyright licence.

**Figure 3:**
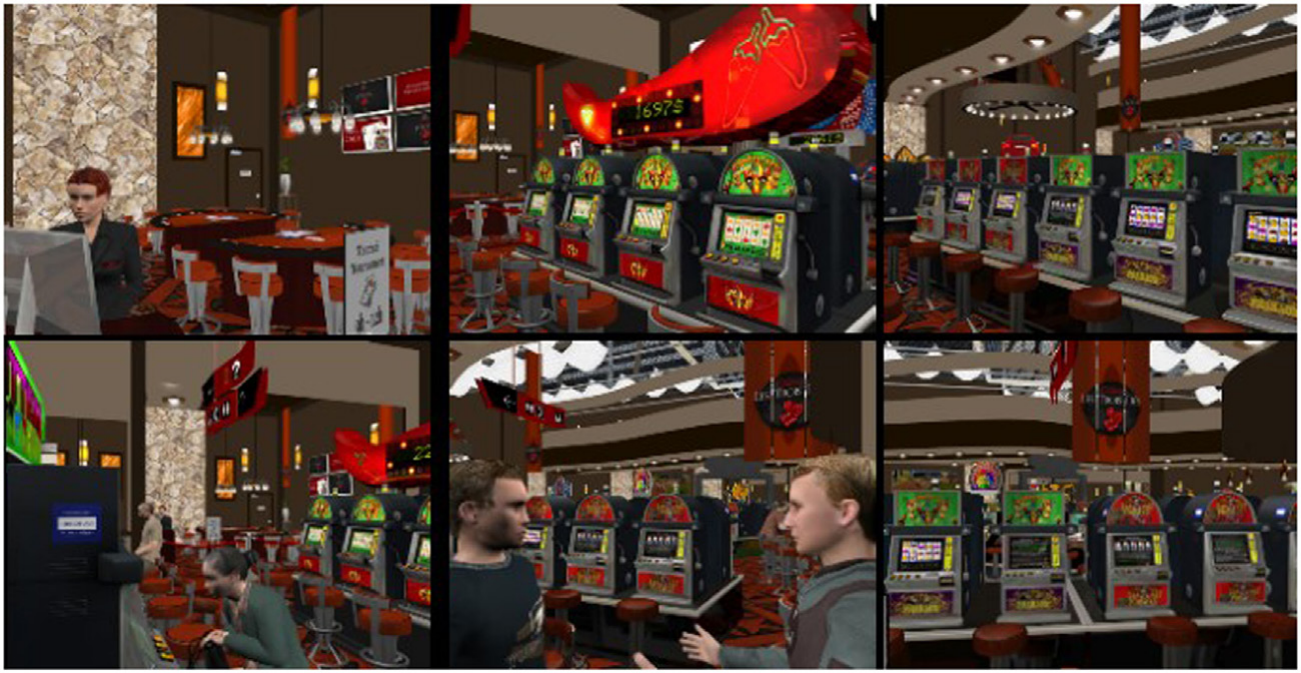
**Illustrations (screenshots) of *The 3Dice* virtual environment used in all three studies**. Reproduced from Bouchard et al. (18) under the Creative Commons copyright licence.

For the experiment, participants signed an informed consent form and were invited to play each of the following games for 7 min: (a) Scrabble™ (control condition), (b) a real VLT with participants gambling $20, (c) a virtual bar with VLTs called *At Fortunes*, and (d) a virtual casino called *The 3Dice* (see Figures 1–3 describing the experimental setup and the virtual environments). The Gambling Craving Scale (GCS) (25) was administered after each game, and each of its subscales will be examined separately: the Anticipation that gambling will be fun (Cronbach’s alpha = 0.84), the urgent Desire to gamble (Cronbach’s alpha = 0.81), and the expectation that gambling will provide relief from negative affect (Cronbach’s alpha = 0.85). For a detailed description of the virtual environments and the VR technology used, see Bouchard et al. (18). The sequence of participation in each condition was randomly distributed. The immersions in VR were conducted using an nVisor SX head-mounted display and a CUBE^2^ motion tracker.

The data were analyzed with two conditions (frequent gamblers experimental condition vs. occasional gamblers control condition) by four repeated measures (Scrabble™—game of skill control condition, real VLT control condition, immersion in the *At Fortunes* virtual bar with VLTs experimental condition, immersion in the *The 3Dice* virtual casino experimental condition) ANOVAs conducted separately with each subscale of the GCS. A Bonferroni correction was applied to control for inflation of the error rate due to multiple comparisons (i.e., alpha set at 0.016). Statistically significant interactions were followed up by *a priori* planned repeated contrasts with the Scrabble™ game of skill control condition, and effect sizes measured with partial eta-squared are reported. The correlation between severity of GD and urge to gamble after immersions in both virtual environments was explored with Pearson’s correlations.

### Results

Descriptive statistics on the impact of the manipulations on the GCS subscales are reported in Table 1. The results of the repeated measures ANOVAs revealed statistically significant main effects on the Anticipation of fun subscale of the GCS [Time *F*_(3,186)_ = 0.5, ns; Condition *F*_(1,62)_ = 10.87, *p* < 0.001; Interaction *F*_(3,186)_ = 11.53, *p* < 0.001] and the Desire to gamble subscale of the GCS [Time *F*_(3,186)_ = 2.35, ns; Condition *F*_(1,62)_ = 5.51, *p* < 0.01; Interaction *F*_(3,186)_ = 4.97, *p* < 0.01]. No significant main effect was found on the Relief from negative affect subscale of the GCS (all *F* < 2.9, ns). The significant repeated measures ANOVAs were followed up by interaction contrasts comparing each exposure to a gambling situation (i.e., real VLT, VR with *At Fortunes*, and VR with *The 3Dice*) with scores following the control condition (i.e., playing Scrabble™). For the Anticipation subscale, all three interaction contrasts were statistically significant [control vs. real VLT *F*_(1,62)_ = 16.31, *p* < 0.001, partial eta-squared = 0.21; control vs. VR with *At Fortunes F*_(1,62)_ = 11.48, *p* < 0.01, partial eta-squared = 0.16; and control vs. VR with *The 3Dice F*_(1,62)_ = 18.3, *p* < 0.001, partial eta-squared = 0.23]. A similar pattern was observed for the Desire subscale [control vs. real VLT *F*_(1,62)_ = 10.97, *p* < 0.001, partial eta-squared = 0.15; control vs. VR with *At Fortunes F*_(1,62)_ = 5.06, *p* < 0.05, partial eta-squared = 0.08; and control vs. VR with *The 3Dice F*_(1,62)_ = 9.96, *p* < 0.01, partial eta-squared = 0.14]. In short, the results suggest that, for “frequent” gamblers, gambling on a real VLT or in VR is associated with a significant increase in anticipation and desire to gamble, which were significantly higher than in very occasional gamblers. In addition, urges to gamble measured with the total score of the GCS post immersion in VR were significantly correlated with the severity of gambling addiction as measured with the SOGS (*r* = 0.49, *p* < 0.001 in *At Fortunes* and *r* = 0.49, *p* < 0.001 in *The 3Dice* for the Anticipation subscale; *r* = 0.47, *p* < 0.001 in *At Fortunes* and *r* = 0.49, *p* < 0.001 in *The 3Dice* for the Desire subscale; and *r* = 0.63, *p* < 0.001 in *At Fortunes* and *r* = 0.38, *p* < 0.001 in *The 3Dice* for the Relief subscale).

**Table 1:** **Mean (and SDs) Gambling Craving Scale (GCS) after playing a control game (Scrabble™) or gambling on a real video lottery terminal (VLT) or in two virtual environments in Study 1**.

GCS subscales	Scrabble™		Real VLT		Virtual reality (VR) *At Fortunes*		VR *The 3Dice*	
	Occasional gamblers	Frequent gamblers	Occasional gamblers	Frequent gamblers	Occasional gamblers	Frequent gamblers	Occasional gamblers	Frequent gamblers
Anticipation of fun	10.47 (6.30)	10.29 (4.17)	7.39 (4.46)	12.68 (5.27)	8.61 (4.25)	12.12 (5.07)	7.22 (4.46)	12.5 (5.29)
Desire to gamble	5.92 (3.95)	6.46 (5.36)	3.94 (2.33)	7.18 (5.32)	4.44 (2.42)	7.32 (5.59)	3.64 (1.42)	6.71 (4.84)
Relief from negative	4.0 (3.25)	4.54 (4.51)	3.0 (0)	4.46 (4.43)	3.08 (0.5)	4.86 (5.37)	3.06 (0.23)	4.36 (4.39)

In summary, the results from Study 1 show that VR can be used to induce cravings in gamblers. Following immersions in VR, the magnitude of the increase on two subscales out of three on the measure assessing the urges to gamble corresponds to large effect sizes that are essentially in the same range as playing on a real VLT. The strong correlations between urges to gamble and the SOGS suggest that results might be generalizable to more severe gamblers, although this remains to be tested empirically. The next step is to test a preliminary treatment protocol with minimal involvement of VR and documents its safety with people suffering from GD.

## Study 2

### Participants

An initial pilot study was conducted to document the potential clinical usefulness of two VR immersions in the treatment of GD. The sample comprised 34 participants suffering from pathological gambling as defined in the DSM-IV-TR (26) and registered at one of two inpatient treatment centers: the CASA Centre (Saint-Augustin-de-Desmaures, QC, Canada) and La Maison L’Odyssée (Sainte-Marie-de-Beauce, QC, Canada). The sociodemographic characteristics of the study’s participants were as follows: 35% women, 87% Canadian, and 14% Natives Americans, median age of 45 (SD = 12.6), average SOGS score of 11.5 (SD = 4.6), average number of days per month spent gambling estimated by the participants at 12.6 (SD = 6.8), number of uncontrollable gambling episodes per month estimated by participants at 9.79 (SD = 8.65), and average amount of money (in Canadian dollars) sunk into gambling per month was estimated by gamblers at $3,710 (SD = $4,963).

Following a random assignment, 14 participants received a traditional 28-day cognitive behavioral treatment program with 2 imaginal exposure exercises (imagination condition), and 20 received the same treatment program but with the 2 exposure exercises conducted using VR immersion (VR condition). A few minimal exclusion criteria were applied during the recruitment of participants: suffering from GD but not associated with VLT or casino slot machines, being a minor, suffering from health issues that could be exacerbated by treatment using VR (major cardiac disorder, severe and frequent motion sickness when traveling by car, vestibular or inner ear disorders, recurring migraines, epilepsy, balance or ocular disease) and suffering from a potentially contraindicated mental health issue (schizophrenia, mental retardation, etc.).

### Method

The two treatment centers taking part in the project have been applying the assessment and treatment program for excessive gamblers developed by Ladouceur et al. (27, 28). When this program is administered in a 28-day inpatient setting, the very first session is devoted to identifying situations that increase the patient’s risk of gambling and the dysfunctional beliefs that support the maintenance of gambling issues. Another session, in the last week of therapy, focuses on practising relapse prevention skills. During both of these sessions, gamblers are asked to imagine gambling situations that trigger cravings and relive these experiences for about 20 min. The intervention that took place during the two gambling exposure sessions (imagination and VR conditions) was recorded in an audio file, and a random selection of 20 of the 59 recordings available (9 sessions were not recorded due to technical problems) were reviewed to confirm that the interventions were conducted as planned. The immersions in VR were performed using a Vuzix iWear VR920 and a CUBE^2^ motion tracker. Before and after the first gambling exposure, the participants were asked to list all of their personal high-risk thoughts and situations. Before and after the second gambling exposure session, participants evaluated the intensity of their desire to gamble on a scale of 0 to 10 and filled out the Simulator Sickness Questionnaire (SSQ) (29, 30). Before and after the treatment program, the participants completed the GCS (25). A few months after the end of the data collection with the participants, focus group-type interviews lasting 1 h 45 min were conducted with the four therapists who carried out the interventions at each center during the data collection (all females staff members of the centers, with a bachelor’s degree in psychology or social sciences, several years of experience with GD, and 2 h of training on the use VR). The goal of the interview was to get their impressions on the VR immersion and the clinical issues they observed.

The usefulness of VR was documented through (a) a detailed review of the content of the sessions and comparison with a Student’s *t*-test of the number of dysfunctional thoughts and high-risk situations identified during the session, (b) a description of the impact of VR immersions on cybersickness using descriptive statistics and a comparison from pre to post immersion using a non-parametric test for comparing means (a Wilcoxon *Z* was used because SSQ scores were not normally distributed), and (c) preliminary data on the impact of treatment using VR were analyzed with 2-condition (VR experimental condition vs. imagination control condition) and 2-time (pre and post-treatment) repeated measures ANOVAs and a multiple regression using residualized change scores.

### Results

A review of the audio recordings revealed that therapists were more inclined to ask patients to express their thoughts and emotions out loud in VR (93 occurrences in the course of treatment) than in imagination (46 occurrences, Chi-square = 15.89, *p* < 0.001). As illustrated in Table 2, VR immersion helps therapists identify more high-risk situations than imaginal exercises. VR also helps to identify twice as many dysfunctional thoughts, but this difference is not statistically significant. Note, however, that the effect size is medium, and that a sample of about 80 participants would provide a 0.80 power to detect a significant difference.

**Table 2:** **New clinical information gathered post therapy session about high-risk situations and dysfunctional thoughts about gambling in Study 2**.

Variable	Condition	Mean	SD	*t*	Eta-squared
Number of high-risk situations reported post-session that were not reported prior to the session	Virtual reality (VR)	2.05	2.01	2.48*	0.17
	Imagination	0.05	1.00		
Number of dysfunctional thoughts reported post-session that were not reported prior to the session	VR	1.53	2.25	1.61	0.08
	Imagination	0.42	1.00		

*p < 0.025.

The number of negative unwanted side effects induced by the immersion in VR in the VR condition was measured before and after the second VR therapy session. The results presented in Table 3 show that the immersion did not lead to an increase in intensity of cybersickness symptoms compared to what was recorded before the immersion (Wilcoxon *Z* test = 0.57, 0.30, and.0, respectively, all ns).

**Table 3:** **Unwanted negative side effects induced by the immersion in virtual reality (VR) (i.e., cybersickness) as measured by the Simulator Sickness Questionnaire (SSQ) in Study 2 before and after the session devoted to relapse prevention**.

	Before the immersion in VR				After the immersion in VR			
SSQ raw score	Mean	SD	Min.	Max.	Mean	SD	Min.	Max.
Total	1.43	1.90	0	6	1.69	3.4	0	13
Nausea subscale	0.56	0.81	0	3	0.56	1.51	0	6
Oculomotor subscale	0.87	1.54	0	5	1.13	2.06	0	7

As preliminary data on the effectiveness of the program with a minimal use of VR, a repeated measures ANOVA was conducted for the total GCS score. Results show a large reduction in cravings in participants in the VR condition, from a mean of 28.00 (SD = 16.9) to a mean of 12.69 (SD = 6.66), and a similar reduction in the control imagination condition, from a mean of 23.62 (SD = 14.78) to a mean of 10.88 (SD = 2.75). The reduction was significant for both conditions, with no differences in terms of treatment modality [Time *F*_(1,19)_ = 14.23, *p* < 0.001; Condition *F*_(1,19)_ = 0.61, ns; Interaction *F*_(1,19)_ = 0.12, ns]. The effect size of the interaction, as assessed with the partial eta-squared, was 0.006. A regression using residualized change scores was also conducted to document the relationship between the intensity of the cravings induced during the relapse prevention session and pre-to-post-treatment improvements on the total score of the GCS. The regression equation was statistically significant [*F*_(2,20)_ = 9.01, *p* < 0.01, Adj *R*^2^ = 0.46], with the increase in cravings during the relapse prevention session being significantly related to more improvement at the end of the program (*t* = 4.19, *p* < 0.001, sr^2^ = 0.48).

The focus group confirmed that therapists were satisfied with the use of VR. No adverse event was reported on the evenings following the therapy sessions where VR was used. Table 4 lists the advantages of using VR as reported during the focus groups. The therapists also made several suggestions that contributed to improving the treatment program and the development of two additional modules where VR could be used for cognitive restructuring (18).

**Table 4:** **Overview of the therapists’ opinions in Study 2 regarding the advantages of using virtual reality immersions**.

Access to spontaneity of patients who are too rational. Easier access to patients’ emotions. Getting around denial by stating contradictions between what is expressed by the patients and how they behave during the immersion. Helps to validate what is learned in therapy and reinforce personal self-efficacy. Allows for the observation of physical reactions associated to cravings. Helps identify intervention cues for other addictions. Provides easier access to erroneous thoughts. Helps to validate patients’ comprehension of therapeutic concepts learned in therapy. Brings patients back to reality, whether they are too confident or not enough.

Overall, the results of Study 2 revealed that therapists can use VR in a clinical setting and that this technology could be useful in eliciting clinically relevant information about patients’ thoughts, behaviors, and high-risk situations. The second session where VR was used revealed that it was not associated with significant cybersickness and that the induction of cravings during the relapse prevention exercise was related to the treatment outcome. Finally, dedicating two sessions to VR instead of following the standard treatment program was not associated with a reduction in treatment effectiveness (see the general discussion for more on VR vs. the standard procedure). VR can therefore be used more intensively in the CBT of GD. However, concerns about the safety of inducing cravings in sessions can be addressed much further.

## Study 3

### Participants

Study 3 primarily aims to document the safety, in terms of intensity of gambling cravings post-session, of applying VR to CBT, and to provide pilot data on increasing the use of VR to four sessions. A sample of 25 adults with a primary diagnosis of GD according to the DSM-5 criteria (1) was recruited following a semi-structured telephone interview conducted by mental health and GD care professionals. The control condition comprised 11 participants; 14 participants were in the experimental condition. Participants were recruited and treated at two GD treatment centers: Centre CASA (St-Augustin-de-Desmaures, QC, Canada) and Maison Jean-Lapointe (Montreal, QC, Canada). The two centers taking part in the project applied the Evaluation and Treatment Program for Excessive Gamblers by Ladouceur et al. (27, 28) with group therapy sessions combined with four individual sessions (i.e., the four targeted for applying VR) in their 28-day inpatient treatment program. The sociodemographic characteristics of the participants were as follows: 50% women, 100% Canadians, mean age of 47 (SD = 12.8), average Canadian Problem Gambling Index (CPGI) score of 19.96 (SD = 3.7), average number of diagnostic GD symptoms encountered during the interview of 7.44 (SD = 1.6), average number of hours per week spent gambling estimated by the participants at 19.9 (SD = 16.95), and weekly average amount lost gambling estimated by gamblers at $1,131 (SD = $1,190).

### Method

As in Study 2, the participants received inpatient treatment from the therapists, but this time four CBT sessions were dedicated to VR immersion [for a detailed treatment manual, see Ref. (18)]. The participants were randomly assigned to the VR stimuli (VR-S) condition or to a control imagination stimuli (Imag-S) condition. To balance out the potential effects of being immersed in VR (e.g., cybersickness), the four treatment sessions for the control Imag-S condition were conducted in VR, but the content of the virtual environment was not associated in any way with gambling or with the induction of gambling cravings. Instead of using the *At Fortunes* bar or *The 3Dice* casino, participants in the control Imag-S condition were immersed in an environment representing an empty room, with no cues associated with gambling or money. Once immersed in this environment, participants were invited to imagine themselves in a high-risk situation and apply the CBT techniques.

The VR sessions relied on the same equipment as that the one used in Study 2 and were dedicated to the identification of high-risk situations, cognitive restructuring, and relapse prevention. Audio recordings of the therapy sessions were played back to confirm that the instructions in the treatment manual were followed. All participants were immersed in VR, with 100% of the participants in the VR-S condition being exposed to virtual gambling cues only, and 100% of the participants in the Imag-S condition being exposed to imaginal stimuli only. The integrity scores regarding compliance with the treatment manual (86.7% for the VR-S condition and 82.5% for the Imag-S condition) confirm that the therapists carried out the interventions as planned. The compliance scores for respecting the clinical objectives of each session were even higher (91.9% for the VR-S condition and 96.9% for the Imag-S condition), which is excellent.

To document the safety of immerging people suffering from GD in VR to elicit cravings over four sessions, participants filled out the My Treatment, an in-house questionnaire administered immediately after the session, and then 12, 24, and 36 h post-session. Five questions were asked (see Table 5), including two of particular interest to us since they measure the intensity of cravings: (a) “In terms of percentage, how much did you feel an urge to gamble?” and (b) “How often did you feel an urge to gamble?” The second goal of Study 3 was to provide pilot data on the impact of a program comprising four CBT sessions conducted as part of VR immersions on three indices of effectiveness, administered before and 2 weeks after the treatment program: (a) the Canadian Problem Gambling Index (CPGI) (31, 32), (b) the number of GD diagnostic criteria according to the Diagnostic Interview for Gambling (DIG) (27, 28), and (c) the Gambling Related Cognitions Scale (GRCS) (33).

**Table 5:** **Mean (and SD) on the My Treatment Questionnaire immediately after each therapy session in Study 3**.

Items	Session #1		Session #2		Session #3		Session #4	
	VR-S	Imag-S	VR-S	Imag-S	VR-S	Imag-S	VR-S	Imag-S
I felt the urge to gamble: %	21.47 (31.51)	30.83 (35.02)	19.29 (26.74)	19.23 (22.62)	15.36 (20.61)	25.91 (34.99)	8.57 (19.16)	13.00 (28.76)
I can control my urge to gamble: %	72.65 (22.02)	57.50 (39.11)	76.07 (19.82)	77.31 (21.76)	73.21 (26.36)	66.82 (35.73)	84.07 (26.32)	93.33 (9.61)
I can control my gambling behaviors: %	57.06 (35.71)	50.42 (39.11)	71.07 (30.52)	48.69 (44.73)	71.79 (26.79)	39.09 (44.09)	80.86 (26.68)	68.33 (40.86)
I think the probability of winning is related to luck: %	82.35 (29.05)	67.50 (41.81)	84.29 (32.75)	93.08 (17.02)	88.57 (28.79)	93.18 (17.93)	97.86 (5.79)	97.50 (8.66)
I felt the urge to gamble: frequency	0.94 (1.44)	1.58 (2.71)	0.57 (0.85)	0.69 (0.86)	0.64 (0.93)	1.27 (1.62)	0.31 (0.48)	0.25 (0.45)

*VR-S, experimental condition where the urge is induced by stimuli recreated in virtual reality; Imag-S, control condition where the urge is induced by stimuli evoked in imagination*.

A descriptive approach was adopted for the data documenting side effects, accompanied by Student’s *t*-tests and two conditions (VR with virtual stimuli associated with gambling experimental condition and VR with imaginal stimuli associated with gambling control condition) by four times repeated measures ANOVAs. Data were analyzed with the goal of documenting the safety of using VR in CBT for GD, and therefore no correction was applied to control the potential inflation of error rate associated with multiple comparisons for assessing side effects. To provide pilot data on the efficacy of replacing standard CBT exercises for GD with the exercise performed in (only) four immersions in VR, 2-condition by 2-time (before treatment and 2 weeks after treatment) repeated measures ANOVAs were conducted using three outcome measures. Effect sizes are reported, Bonferroni corrections applied (i.e., alpha set at 0.016) and intent-to-treat data were used to remain conservative.

### Results

The issue of post-session intensity of the desire to gamble induced in VR was studied through a review of the questionnaires filled out by the participants after each of the four therapy sessions in which therapists induced the urge to gamble through either VR or imaginal exercises (Tables 5–7). *T* tests were performed to compare the two conditions based on all of the variables measured immediately following the four therapy sessions. The only statistically significant difference (*t* = 2.29, *p* < 0.05) regarded the impression that the treatment provided helped better control gambling issues, as completed following the third session (i.e., the last cognitive restructuring session) for participants in the VR-S condition. No other comparison^1^ on the data reported in Table 5 even came close to the significance threshold. This shows that VR does not induce an urge to gamble that persists post-session longer or more strongly than after imaginal therapy; that the urge remains low; and that the impression of being able to control the urge remains high.

Tables 6 and 7 show how the urge to gamble becomes more or less intense in the hours following the four sessions. A repeated measures ANOVA reveals a decline in the evaluated percentage in the first session (Table 6) over time [*F*_(3,75)_ = 4.01, *p* < 0.025], no difference between conditions [*F*_(1,25)_ = 0.08, ns], and no significant interaction [*F*_(3,75)_ = 0.48, ns]. Once again we see a significant drop over time during the second [*F*_(3,69)_ = 2.86, *p* < 0.05] and third [*F*_(3,63)_ = 6.11, *p* < 0.001] therapy sessions. The conditions and interactions effects are not significant. It is of interest to note that the urge to gamble in the hours and days following the sessions levels off without ever dropping down to zero, most likely pointing to the everyday degree of desire in the gamblers being treated.

**Table 6:** **Mean (and SD) of the urge to gamble measured in percentage by My Treatment Questionnaire after therapy sessions and after 12, 24, and 36 h in Study 3**.

Session	Post		12 h		24 h		36 h	
	VR-S	Imag-S	VR-S	Imag-S	VR-S	Imag-S	VR-S	Imag-S
#1	21.47 (31.51)	31.00 (36.04)	13.65 (25.91)	14.50 (22.91)	10.65 (23.55)	12.00 (18.74)	9.47 (20.43)	6.00 (9.66)
#2	19.29 (26.74)	14.09 (16.56)	9.36 (20.14)	6.36 (15.02)	8.64 (15.08)	5.91 (10.68)	5.79 (8.47)	6.82 (11.89)
#3	15.36 (20.61)	22.78 (32.70)	1.50 (3.61)	1.67 (3.54)	6.50 (16.43)	2.22 (4.41)	7.93 (15.74)	6.67 (14.14)
#4	9.23 (19.77)	14.60 (31.44)	0.77 (2.77)	16.00 (32.39)	0.85 (2.76)	12.50 (31.20)	3.08 (7.51)	13.00 (31.29)

*VR-S, experimental condition where the urge is induced by stimuli recreated in virtual reality; Imag-S, control condition where the urge is induced by stimuli evoked in imagination*.

**Table 7:** **Mean (and SD) of the urge to gamble measured in frequency by My Treatment Questionnaire after therapy sessions and after 12, 24, and 36 h in Study 3**.

Sessions	Post		12 h		24 h		36 h	
	VR-S	Imag-S	VR-S	Imag-S	VR-S	Imag-S	VR-S	Imag-S
#1	0.94 (1.44)	1.00 (1.49)	1.19 (2.07)	0.90 (1.29)	0.75 (1.29)	0.90 (1.66)	0.69 (1.25)	0.80 (1.87)
#2	0.54 (0.88)	0.45 (0.52)	0.38 (0.65)	0.36 (0.67)	0.54 (0.78)	0.55 (0.93)	0.54 (0.88)	0.73 (0.91)
#3	0.64 (0.93)	1.22 (1.79)	0.14 (0.36)	0.11 (0.33)	0.36 (0.63)	0.33 (0.50)	0.57 (1.16)	0.89 (1.54)
#4	0.33 (0.49)	0.30 (0.48)	0.08 (0.29)	0.30 (0.68)	0.08 (0.29)	0.20 (0.42)	0.33 (0.78)	0.10 (0.32)

*VR-S, experimental condition where the urge is induced by stimuli recreated in virtual reality; Imag-S, control condition where the urge is induced by stimuli evoked in imagination*.

As regards frequency of the urge to gamble (Table 7) after the sessions, none of the ANOVAs revealed a significant effect except for the time effect following the third therapy session [*F*_(3,63)_ = 2.91, *p* < 0.025]. Overall, the low frequency of episodes of gambling cravings might be indicative of a low urge to gamble following the sessions but likely also reflects a difficulty retrospectively isolating multiple distinct episodes. As such, someone who would constantly feel the urge to gamble throughout the day may report just one episode or at most a few distinct episodes if attention was distracted away from a constant craving. A percentage assessment of the entire day is probably more accurate than the frequency method.

Repeated measures ANOVAs were performed for the three effectiveness measurements (see Table 8). The results show large effect sizes (partial eta-squared of.46, 0.9, and 0.85, respectively) and statistically significant improvement in the three measures. Analyses revealed no significant difference with regard to the time × condition interactions, with effect sizes ranging from small (0.001) for the CPGI, medium for the number of diagnostic criteria encountered with the DIG (0.07), and dysfunctional beliefs as measured using the GRCS (0.04). We can thus estimate that with 0.80 power, some 60 participants per condition would be needed for these interactions to be statistically significant. Using a cut-off score of 7 or less for the CPGI post-treatment (i.e., the cut-off score for GD), we get a 50% success rate for VR-S and a 45.5% success rate for the Imag-S control condition. Using a cut-off score of 4 or less for the DIG (i.e., the number of diagnostic criteria required to receive a DG diagnostics), the success rate is 56% for the VR-S condition and 44% for the Imag-S control condition. These differences are not statistically significant. Such preliminary results accordingly point not only to the success of the VR-S program but also underscore the need to pursue more research to increase its short-term effectiveness on GD symptoms.

**Table 8:** **Mean (and SD) on efficacy measures pre- and post-treatment in Study 3**.

Measures	Pre		Post 2 weeks		Repeated measures ANOVA		
	VR-S	Imag-S	VR-S	Imag-S	Time *F*_(1,23)_	Cond *F*_(1,23)_	Interaction *F*_(1,23)_
CPGI	19.86 (3.84)	20.09 (2.55)	11.21 (9.64)	10.82 (8.32)	19.62***	0.002	0.02
DIG (*n*. Dx criteria)	7.00 (1.96)	8.00 (0.82)	1.29 (1.20)	1.10 (1.66)	193.08***	0.91	1.71
GRCS-total	81.36 (27.09)	87.18 (24.33)	30.07 (7.62)	26.18 (4.33)	131.69***	0.03	0.99

Intent-to-treat data, ***p < 0.001.

*VR-S, experimental condition where the urge is induced by stimuli recreated in virtual reality; Imag-S, control condition where the urge is induced by stimuli evoked in imagination; CPGI, Canadian Problem Gambling Index; DIG, Diagnostic Interview of Gambling (number of diagnostic criteria met by the participant); GRCS, Gambling Related Cognitions Scale*.

Essentially, the results of Study 3 illustrate three phenomena: (a) there is no difference in lasting effects on the urge to gamble between the sessions where VR-S were applied and imaginal stimuli were used; (b) the post-session urge to gamble is comparable across all conditions and hovers around 20% immediately following the therapy sessions for participants in the VR-S and the Imag-S conditions; and (c) the use of only four VR sessions in CBT for GD can lead to success rates between 50 and 56% and medium effect sizes.

## Discussion

This series of three studies helps determine the potential and safety of VR for the treatment of GD. The studies show that it is possible to induce a significant urge to gamble, as strong as that observed using a real VLT. A gradual progression in the number of sessions while keeping a watchful eye on potential adverse effects sets the stage for a more intensive use of VR with GD. This technology was well accepted and used by our therapists, helping them work with patients’ who are more emotionally aroused during therapy sessions. It also helps identify elements that are useful for the therapy, namely high-risk situations. Cravings are induced at levels that are easily manageable by therapists during the sessions and that are not a cause for concern post-session. That does not mean that post-session cravings can be ignored completely, but they are certainly not overly worrisome. Of course, it is up to each therapist to be well informed and know how to handle these cravings with his or her patients. The unwanted negative side effects induced by immersions in VR seem minor. The project provides all of the information necessary to initiate a large-scale clinical trial and increase the number of sessions incorporating VR immersions in CBT strategies to more than four sessions.

The results essentially open the way for a new approach to the treatment of GD. This technology opens the door to powerful new prevention and treatment tools for therapists that will also appeal to gamblers. Yet, therapists and case workers may still question the potential of VR to induce cravings because gamblers make no actual monetary gains when gambling in VR. The demonstration in this paper that an immersion in VR can stimulate the urge to gamble in gamblers builds on the work done by Kushner et al. (34) and Wulfert et al. (35), which showed that gambling urges could be induced by stimuli in a laboratory, and research from Young et al. (36) demonstrating that gambling urges could be influenced, even in VR.

Through a number of pilot cases, research studies from Garcia-Palacios et al. (22), Giroux et al. (19), and Park et al. (20) point to VR’s potential as a clinical tool. The data obtained from the current project provide a solid empirical basis justifying a number of new research projects as well as large-scale randomized control clinical trials, such as (a) comparing the effectiveness of immersive and non-immersive versions (i.e., using only the computer screen rather than a head-mounted display); (b) distinguishing cybersickness symptoms from the physical signs of gambling cravings and withdrawal; (c) examining the role played by the sense of presence in VR immersions; (d) using virtual stimuli associated with other addictions (e.g., presence of alcohol in the virtual environment) to see their impact on GD therapy; (e) clarifying the impact of environmental factors (e.g., bank machine) on the urge to gamble; (f) using VR with other forms of psychotherapy, including mindfulness; (g) evaluating how using VR can boost the patient’s motivation in therapy; and (h) conducting research on potential VR addiction, an as-yet non-existent phenomenon but clearly one that should be monitored closely.

The fact that VR was not more effective than the control condition raises the question of the relevance of using this technology. There are several elements to consider in this regard. First, the success rates of using VR in CBT were far from inferior to standard CBT, a finding that mimics what was found in the first studies on using VR in the treatment of anxiety disorders and led to a now-flourishing field of useful clinical applications [for a review, see Ref. (37)]. Indeed, it took several trials with people suffering from specific phobia showing that VR was not more or less effective than *in vivo* exposure to develop treatment protocols that fully exploit the potential of VR and make it more effective. Second, CBT was applied using virtual craving stimuli in only very few sessions. Dedicating only two sessions to cognitive restructuring and one to relapse prevention is likely insufficient to exploit the full potential of VR. Now that safety seems sufficiently documented, as many sessions of VR as possible should be integrated in the treatment protocol to really tests if VR can be more effective than the standard procedure. Third, the interventions were integrated in routine 28-day inpatient programs, with much less control in terms of content than what can be found in randomized control trials. It is difficult to isolate in the two pilot trials presented in this article the contribution of each specific intervention. Also, strong follow-up data are required to fully comment on the efficacy of the interventions. Finally, the advantages of VR should not be examined only in terms of the reduction of symptoms but also in terms of motivation to attract and retain patients in treatment programs, effort by the therapists, and therapists’ motivation to actually use exposure to gambling cues (either in imagination or in real-life settings).

Until now, given the purchase cost of the equipment involved, only well-funded research centers had access to VR. But with the advent of a number of hardware companies aiming for the mass market, such as Oculus™ (owned by Facebook), Vive™ (owned by HTC), GearVR™ (owned by Samsung), and Google Cardboard™ (owned by Google), just to name a few, VR will quickly become a mass market product, and applications are now available to gamble real money while immersed in virtual casinos. The advantage with VR applications developed for clinicians over those developed for the gaming industry is that people with GD cannot use them outside the therapist’s office to fuel their addiction. The arrival of VR brings with it all of its advantages, namely as regards to availability of new psychotherapeutic tools, and its disadvantages, such as the risk of addiction to various non-therapeutic applications that make it possible to escape the challenges of day-to-day life or gamble from the comfort of the patient’s own home.

## Author Contributions

All authors listed have made substantial, direct, and intellectual contribution to the work and approved it for publication.

## Conflict of Interest Statement

SB and GR are consultants to and own equity in Cliniques et Développement In Virtuo, which develops virtual environments. The remaining authors report no financial relationship with commercial interests.

## Footnotes

^1^Results not reported but available upon request.
